# Endoscopic resection for local residual or recurrent cancer after definitive chemoradiotherapy or radiotherapy for esophageal squamous cell carcinoma

**DOI:** 10.1038/s41598-023-32667-5

**Published:** 2023-06-28

**Authors:** Yasuhiro Tani, Ryu Ishihara, Noriko Matsuura, Yuki Okubo, Yushi Kawakami, Hirohisa Sakurai, Takahiko Nakamura, Katsunori Matsueda, Muneaki Miyake, Satoki Shichijo, Akira Maekawa, Takashi Kanesaka, Sachiko Yamamoto, Yoji Takeuchi, Koji Higashino, Noriya Uedo, Tomoki Michida

**Affiliations:** 1 Department of Gastrointestinal Oncology, Osaka International Cancer Institute, 3-1-69, Otemae, Chuo-ku, Osaka, 541-8567 Japan; 2 Division of Research and Development for Minimally Invasive Treatment, Cancer Center, Keio University School of Medicine, Tokyo, Japan

**Keywords:** Cancer therapy, Gastrointestinal cancer

## Abstract

Chemoradiotherapy (CRT) and radiotherapy (RT) are treatment options for esophageal squamous cell carcinoma (ESCC), but local residual/recurrent cancer after CRT/RT is a major problem. Endoscopic resection (ER) is an effective treatment option for local residual/recurrent cancer. To ensure the efficacy of ER, complete removal of endoscopically visible lesions with cancer-free vertical margins is desired. This study aimed to identify the endoscopic parameters associated with the complete endoscopic removal of local residual/recurrent cancer. In this single-center, retrospective study, we used a prospectively maintained database to identify esophageal lesions that were diagnosed as local residual/recurrent cancer after CRT/RT and treated by ER between January 2012 and December 2019. We evaluated the associations of endoscopic R0 resection with findings on conventional endoscopy and endoscopic ultrasonography (EUS). In total, 98 lesions (83 cases) were identified from our database. The rate of endoscopic R0 resection was higher for flat lesions (100% versus 77%, P = 0.00014). EUS was performed for 24 non-flat lesions, and endoscopic R0 resection was achieved for 94% of lesions with an uninterrupted fifth layer. Flat lesions on conventional endoscopy and lesions with an uninterrupted fifth layer on EUS are good candidates for ER.

## Introduction

Esophageal cancer is the seventh most common cancer and sixth most common cause of cancer-related mortality worldwide^1^, and squamous cell carcinoma is the predominant type of esophageal cancer in Asia^2^. Chemoradiotherapy (CRT) is a treatment option for esophageal squamous cell carcinoma (ESCC), and radiotherapy (RT) is applied for patients who are not suitable for CRT, such as elderly patients and patients with complications. Although CRT/RT can preserve the esophagus and is expected to achieve a complete response, local residual/recurrent cancer after CRT/RT remains a major problem^3^. Although esophagectomy is a promising treatment for local residual/recurrent cancer, it is associated with high mortality and morbidity rates^4,5^. Endoscopic resection (ER) is an alternative to esophagectomy that is minimally invasive and effective if complete resection of local residual/recurrent cancer is achieved^6,7^.

The major limitation of ER is difficulty in achieving en bloc R0 resection. Low R0 resection rates have been reported for ER of local residual/recurrent cancer because of its technical difficulty^7,8^. Even if pathological examination of the resected specimen reveals a positive horizontal margin, the outcome after ER is usually favorable if the endoscopically visible lesion is removed^9^. However, the outcome after ER with pathological evidence of a positive vertical margin has not been thoroughly evaluated. To ensure the efficacy of ER, complete removal of the endoscopically visible lesion with a cancer-free vertical margin is desired.

To completely remove the lesion with a cancer-free vertical margin, accurate preoperative assessment of the cancer invasion depth is essential. However, this is sometimes difficult for local residual/recurrent cancer, mainly because of fibrosis and vascular change caused by CRT/RT. To the best of our knowledge, few studies have investigated the accuracy of the preoperative diagnosis for ER of local residual/recurrent cancer after CRT/RT. Therefore, this study aimed to identify the preoperative diagnostic factors associated with the complete endoscopic removal of local residual/recurrent cancer.

## Methods

### Study design and patients

This was a single-center, retrospective study. Using a prospectively maintained database, we identified esophageal cancerous lesions that were diagnosed as local residual/recurrent cancer after CRT/RT and treated by ER between January 2012 and December 2019. The inclusion criteria were as follows: (1) local residual/recurrent cancer after definitive CRT/RT clinically diagnosed as T1 by endoscopy and/or endoscopic ultrasonography (EUS) and (2) pathologically confirmed esophageal cancer using the resected specimen. Locally recurrent tumors were defined as lesions that recurred after the confirmation of complete response to CRT/RT at least once, whereas residual tumors were defined as lesions that did not disappear after CRT/RT. Lesions diagnosed as local recurrence after ER or photodynamic therapy (PDT) were excluded.

### Pretreatment evaluation and treatment strategy

**Figure 1:**
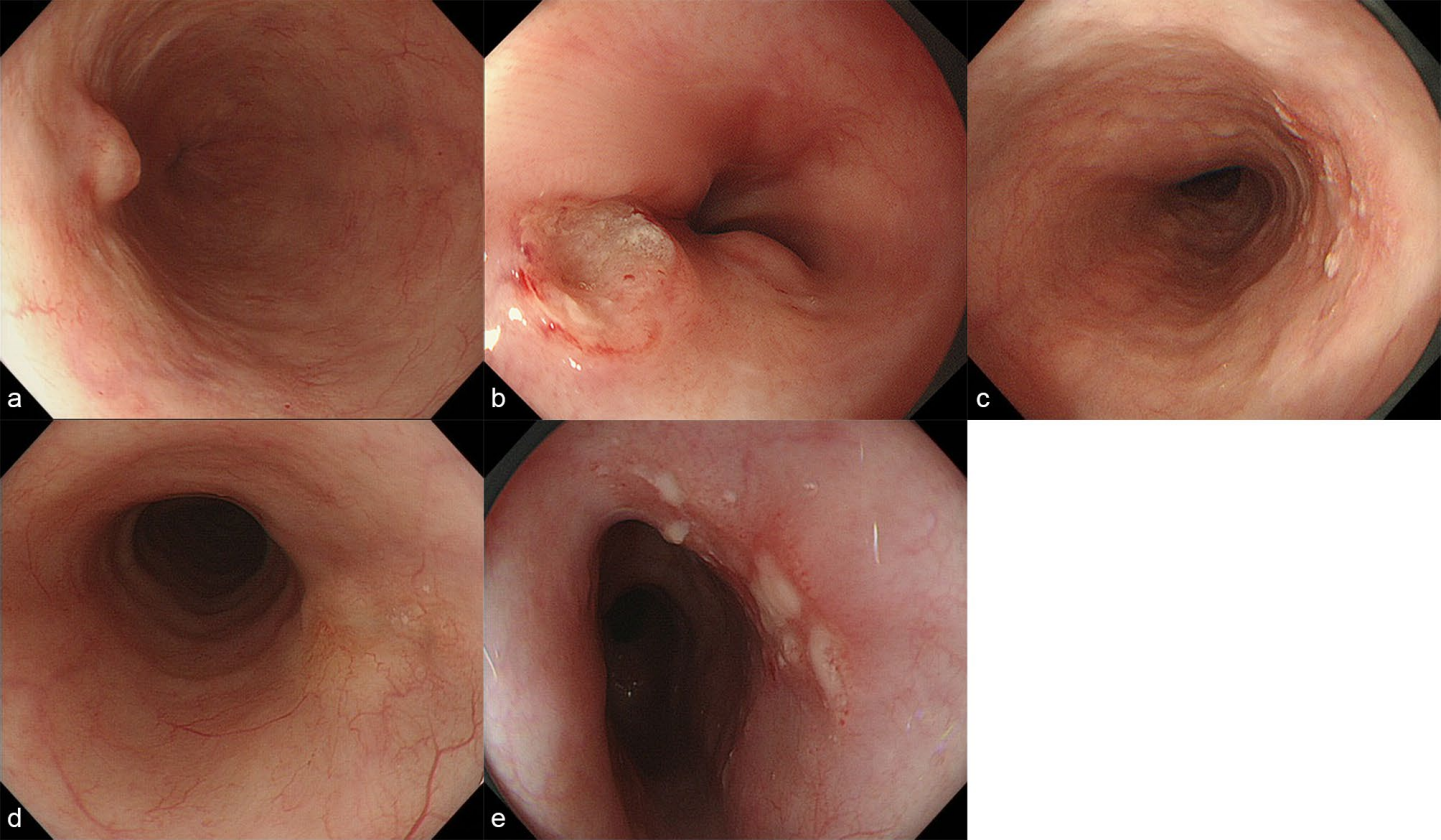
Representative images of endoscopic findings. (**a**) Protrusion. (**b**) Excavation. (**c**) Unevenness. (**d**) Submucosal tumor-like appearance. (**e**) Erosion.

Before treatment, endoscopy was performed with white light imaging, narrow-band imaging, or blue laser imaging, as well as iodine staining. The location, macroscopic type (protrusion, excavation, unevenness, submucosal tumor-like appearance, and erosion), lesion size, and circumference of the lesion were evaluated. Protrusion was defined as the presence of an elevation of > 1 mm, whereas excavation was defined as the presence of a depression of > 1 mm. Lesions with protrusion, excavation, unevenness, submucosal tumor-like appearance, and erosion were defined as non-flat lesions. Representative images of these findings are presented in Fig. 1.

**Figure 2:**
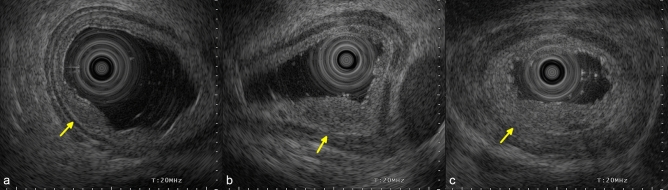
Endoscopic ultrasonography findings of local residual/recurrent cancer after CRT/RT. (**a**) Uninterrupted fifth layer (arrow). (**b**) Slurred fifth layer (arrow). (**c**) Ruptured fifth layer (arrow). CRT, chemoradiotherapy; RT, radiotherapy.

EUS was additionally performed for lesions with endoscopic findings suspicious for submucosal invasion by board-certified fellows of the Japan Gastroenterological Endoscopy Society with extensive experience in EUS of gastrointestinal tract tumors. EUS was mainly performed using a jelly-filled method with a 20-MHz miniature probe, and it typically demonstrated the esophageal wall as a nine-layered structure^10^. The cancer invasion depth and continuity of the fifth layer were evaluated using EUS (Fig. 2).

Computed tomography was usually performed for ESCC staging. ER was principally selected when ESCC was diagnosed as clinical N0M0.

ER was mainly performed for local residual/recurrent T1 cancer (mucosal or submucosal cancer). PDT was mainly performed for cancers diagnosed as suspicious for T2.

### ER

ER was performed via endoscopic mucosal resection (EMR) or endoscopic submucosal dissection (ESD). EMR was performed using the transparent distal translucent cap method^11^. ESD was mainly performed using a HookKnife (Olympus, Tokyo, Japan) or FlushKnife BT (Fujifilm Medical, Tokyo, Japan). In most ESD procedures, the clip with line method was applied to gain good counter traction^12^.

### Histological assessments

Resected specimens were fixed in 10% formalin and cut into 2-mm slices. After the specimens were embedded in paraffin, they were sliced into 3-μm-thick sections and stained with hematoxylin and eosin. Histological assessments were performed according to the Japanese Classification of Esophageal Cancer guidelines^13^. The invasion depth, horizontal depth, and vertical margin were evaluated histologically. R0 resection was defined as en bloc resection with negative margins. Endoscopic R0 resection was defined as endoscopic total removal of the lesion with a negative vertical margin. Endoscopic R1 resection was defined as resection that did not meet the criteria for endoscopic R0 resection.

### Statistical analysis

All continuous variables are reported as the median (range), and all categorical variables are summarized as frequencies (percentages). Fisher’s exact test and the Mann–Whitney U test were used to compare clinical variables. P < 0.05 indicated statistical significance. All analyses were performed on a personal computer using the EZR software package, version 1.55 (Saitama Medical Center, Jichi Medical University, Tochigi, Japan)^14^.

### Ethics approval

Waived informed consent was obtained from the ethics committee of Osaka International Cancer Institute. Instead of obtaining informed consent, all participants were provided opportunities to decline study participation prior to the present investigation using the opt-out method on the website of Osaka International Cancer Institute. The study protocol including opt-out consent was approved by the ethics committee of Osaka International Cancer Institute on March 4, 2022 (No. 21216). This retrospective study was performed in accordance with relevant guidelines and regulations.

## Results

### Patients and lesions

In total, 98 lesions (83 patients) were identified from our database. The characteristics of the patients and lesions are summarized in Table 1. For cancer before CRT/RT, the proportion of clinical T1 lesions was 67%. The radiation dose was unknown in one patient, but he was treated with a radiation dose exceeding 50 Gy as the primary therapy. Of the remaining 82 patients, 100%, 59% and 7% received chemotherapy with radiation doses of at least 50, 60, and 70 Gy, respectively. The chemotherapy regimen was unknown in 4 of 69 patients treated with CRT. Of the remaining 65 patients, 91%, 89% and 94% received chemotherapy with 5-FU, CDDP, and platinum agents including CDDP, respectively. For residual /recurrent cancer, the median tumor size was 12 mm (2–50), and 80% of tumors were recurrent lesions.

**Table 1:** Characteristics of the 83 patients and 98 lesions.

Median age, years (range)	70 (46–90)
Sex, n (%)	
Male/female	67/16 (81/19)
Primary therapy, n (%)	
CRT/RT	69/14 (83/17)
cT stage before CRT/RT, n (%)	
T1/T2/T3/T4/unknown	66/19/5/7/1 (67/19/5/7/1)
Tumor status after CRT/RT, n (%)	
Recurrence/Residual	78/20 (80/20)
Location, n (%)	
Ce/Ut/Mt/Lt	1/23/39/35 (1/23/40/36)
Macroscopic type, n (%)	
Is/IIa/IIb/IIc/combined type	10/6/16/60/6 (10/6/16/61/6)
Median tumor size, mm (range)	12 (2–50)
Circumference, n (%)	
< 3/4/ ≥ 3/4	94/4 (96/4)

CRT, chemoradiotherapy; RT, radiotherapy.

### ER and histopathological assessments

The proportion of lesions treated with ESD was 72% (71/98 lesions). The en bloc and endoscopic R0 resection rates were 94% and 91%, respectively. The endoscopic R0 resection rate was not significantly different between ESD and EMR (89% versus 96%, P = 0.437). The adverse events included two cases of perforation and four cases of esophageal stricture requiring dilatation for symptoms. The characteristics of ER and histopathological assessments are summarized in Table 2. The comparison between flat lesions and non-flat lesions are summarized in Table 3.

**Table 2:** Characteristics of endoscopic resection and histopathological assessments.

Endoscopic resection, n (%)	
ESD/EMR	71/27 (72/28)
Invasion depth, n (%)	
EP/LPM/MM/SM1/SM2/undetermined	31/30/8/4/24/1 (32/31/8/4/24/1)
Histological type, n (%)	
SCC/adenocarcinoma/others	97/0/1 (99/0/1)
Horizontal margin, n (%)	
Negative/positive/unclear	80/5/13 (82/5/13)
Vertical margin, n (%)	
Negative/positive/unclear	89/8/1 (91/8/1)
Lymphatic invasion, n (%)	
Negative/positive	93/5 (95/5)
Venous invasion, n (%)	
Negative/positive	91/7 (93/7)
En bloc resection, n (%)	94 (96)
R0 resection, n (%)	72 (73)
Endoscopic R0 resection, n (%)	89 (91)

EMR, endoscopic mucosal resection; EP, epithelium; ESD, endoscopic submucosal dissection; LPM, lamina propria; MM, muscularis mucosae; SM, submucosal layer.

**Table 3:** Characteristics of endoscopic resection and histopathological assessments in flat lesions and non-flat lesions.

	Flat lesions, n = 59	Non-flat lesions, n = 39	P
Endoscopic resection, n (%)			
ESD/EMR	39/20 (66/34)	32/7 (82/18)	0.11
Invasion depth, n (%)			
EP/LPM/MM/SM1/SM2/undetermined	29/25/3/2/0/0 (49/42/5/3/0/0)	2/5/5/2/24/1 (5/13/13/5/62/3)	< 0.0001
Horizontal margin, n (%)			
Negative/positive/unclear	48/1/10 (81/2/17)	32/4/3 (82/10/8)	0.085
Vertical margin, n (%)			
Negative/positive/unclear	59/0/0 (100/0/0)	30/8/1 (77/21/3)	0.00014
Lymphatic invasion, n (%)			
Negative/positive	59/0 (100/0)	34/5 (87/13)	0.0085
Venous invasion, n (%)			
Negative/positive	59/0 (100/0)	32/7 (82/18)	0.0011
En bloc resection, n (%)	56 (95)	38 (97)	> 0.99
R0 resection, n (%)	46 (78)	26 (33)	0.25
Endoscopic R0 resection, n (%)	59 (100)	30 (77)	0.00014

P < 0.05 was considered statistically significant.

EMR, endoscopic mucosal resection; EP, epithelium; ESD, endoscopic submucosal dissection; LPM, lamina propria; MM, muscularis mucosae; SM, submucosal layer.

### Endoscopic R0 resection and endoscopic and EUS findings

The associations between endoscopic R0 resection and conventional endoscopic findings (e.g., elevation, excavation, unevenness, submucosal tumor like appearance, protuberance within the depression, and erosion) are presented in Table 4. Endoscopic R0 resection was significantly associated with elevation, an excavation submucosal tumor like-appearance, and non-flat lesions. The endoscopic R0 resection rates were 100% (59/59 lesions) for flat lesions and 77% (30/39 lesions) for non-flat lesions (P = 0.00014).

**Table 4:** Associations between endoscopic R0 resection and conventional endoscopic and endoscopic ultrasonography findings.

	Endoscopic R0 resection rate	P
Conventional endoscopic findings		
Protrusion		
No	96% (82/85)	0.00011
Yes	54% (7/13)	
Excavation		
No	93% (86/92)	0.0095
Yes	50% (3/6)	
Unevenness		
No	96% (65/68)	0.022
Yes	80% (24/30)	
Submucosal tumor like appearance		
No	96% (80/83)	0.00031
Yes	60% (9/15)	
Erosion		
No	92% (81/88)	0.23
Yes	80% (8/10)	
Flat lesion	100% (59/59)	0.00014
Non-flat lesion	77% (30/39)	
EUS findings		
Uninterrupted fifth layer	94% (17/18)	0.0065
Slurred or ruptured fifth layer	33% (2/6)	
Uninterrupted or slurred fifth layer	78% (18/23)	> 0.99
Ruptured fifth layer	100% (1/1)	

P < 0.05 was considered statistically significant.

EUS, endoscopic ultrasonography.

EUS was performed for 24 non-flat lesions, and the EUS findings of fifth layer were categorized into the following: uninterrupted (18 lesions), slurred (5 lesions), or ruptured (1 lesion). For lesions treated by CRT, the fifth layer was uninterrupted in 12 lesions, slurred in 3 lesions, and ruptured in 1 lesion. For lesions treated by RT, the fifth layer was uninterrupted in six lesions and slurred in two lesions. There was no significant association between the treatment regimen (CRT or RT) and continuity of fifth layer (P > 0.99). The associations between endoscopic R0 resection and EUS finding are presented in Table 4. There was a significant association between endoscopic R0 resection and the presence of an uninterrupted fifth layer (P = 0.0065). For tumors with an uninterrupted fifth layer, the endoscopic R0 resection rate was 94% (17/18 lesions).

### Surveillance endoscopy and the local control status after ER

Of 83 patients, 25 were excluded from surveillance at our hospital, including 2 patients who underwent esophagectomy (one for a positive vertical margin on resected specimen and the other for residual disease confirmed immediately after ER) and 23 patients who underwent surveillance at a referral source hospital. In total, 71 lesions in 58 patients were subjected to surveillance endoscopy at our hospital. The median time from ER to the last endoscopy and median number of surveillance endoscopies were 51 months (6–126 months) and 11 (2–26), respectively. Local recurrence was observed in 12 patients (12 lesions). Six patients underwent additional ER, and five patients underwent ablation. One patient with a poor general condition did not receive additional treatment. One patient with re-recurrence after additional ER underwent esophagectomy.

### Proposed strategy of preoperative evaluation

**Figure 3:**
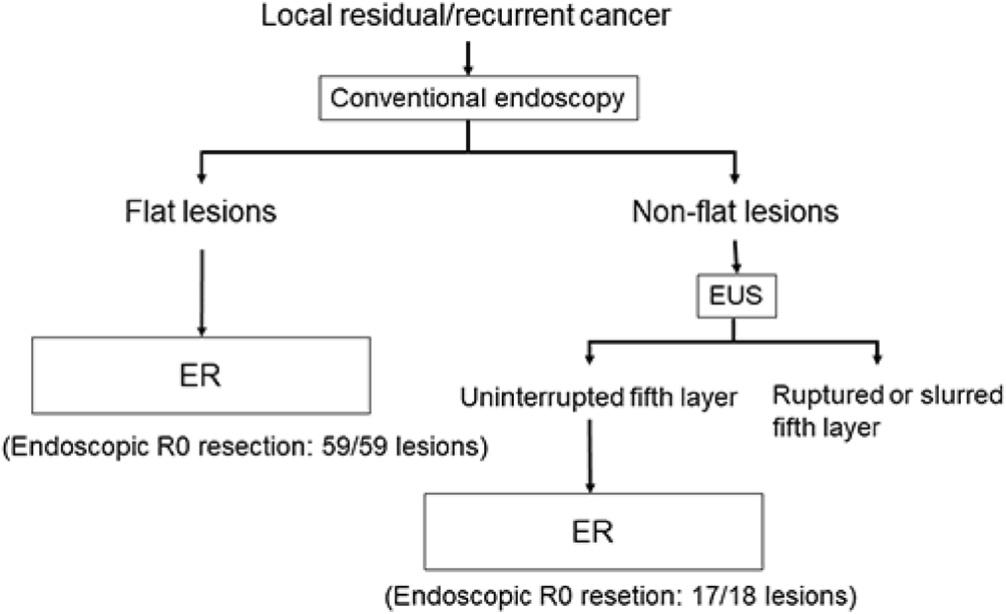
The proposed strategy of the preoperative evaluation for local residual/recurrent cancer after CRT/RT. CRT, chemoradiotherapy; ER, endoscopic resection; EUS, endoscopic ultrasonography; RT, radiotherapy.

Based on the results of this study, we have proposed a strategy for preoperative evaluation (Fig. 3). Flat lesions on conventional endoscopy, which had a 100% endoscopic R0 resection rate, are good candidates for ER. Lesions with an uninterrupted fifth layer on EUS are also good candidates for ER, as indicated by an endoscopic R0 resection rate of 94%.

## Discussion

In this study, the endoscopic R0 resection rate for flat lesions on conventional endoscopy was 100% (59/59 lesions), and that for lesions with an uninterrupted fifth layer on EUS was 94% (17/18 lesions).

EUS is widely used to diagnose T stage ESCC^15–17^. Conversely, a recent multicenter prospective study revealed that additional EUS after conventional endoscopy did not improve the diagnostic accuracy regarding the invasion depth of T1 ESCC^18^. In the diagnosis of local recurrent cancer after CRT/RT, radiation-induced fibrosis can make an accurate diagnosis more difficult compared with that of ordinal esophageal cancer. However, the diagnostic accuracy concerning the cancer invasion depth is not important for the preoperative diagnosis of local recurrent cancer because additional esophagectomy is indicated mainly based on the completeness of the resection. Therefore, the preoperative assessment of resectability is more important than the assessment of the cancer invasion depth in cases of local recurrent cancer after CRT/RT. In this study, we evaluated interruption, slurring, or rupture of the fifth layer by EUS and found that endoscopic R0 resection was achieved in 94% (17/18) of lesions without interruption of the fifth layer, versus 33% (2/6) in lesions with a slurred or ruptured fifth layer. Therefore, EUS might be a useful modality for the preoperative assessment of local residual/recurrent cancer after CRT/RT.

Although the endoscopic R0 resection rate was 91% for all 98 lesions, the R0 resection rate was only 73%. Several possible causes can be considered. Prior CRT/RT can induce scaring and submucosal fibrosis, which makes ER difficult. Furthermore, in cases complicated by stricture induced by prior CRT/RT, a sufficient horizontal margin had to be sacrificed to avoid worsening of the stricture. However, the en bloc and R0 resection rates were comparable to those in previous reports^7,8,19^. Furthermore, the horizontal margin was not a risk factor for failure after ER in a previous study^20^. Therefore, ER can be curative as long as the endoscopically visible lesion is removed with a negative vertical margin.

PDT is a treatment option for local residual/recurrent cancer after CRT/RT, and it is indicated for T1–T2 lesions. Hatogai et al. reported 5-year local failure-free rates of 66.7% for EMR and 51.7% for PDT^6^. Because lesions treated by PDT included more advanced lesions (26.3% were T2 lesions), a strict comparison of efficacy between PDT and ER is difficult. In addition, ER and PDT have different characteristics. Specifically, PDT requires a special device for laser irradiation, and patients must avoid phototoxicity caused by the injection of a photosensitizer. Meanwhile, ER is a more common procedure, but it requires high skill for complete resection. Thus, further evaluation of ER is needed to clarify whether it has advantages over PDT in the treatment of local recurrent cancers after CRT/RT.

In this study, the 98 examined tumors consisted of 59 flat lesions, 39 non-flat lesions, and 18 lesions with an uninterrupted fifth layer. High endoscopic R0 resection rates were observed for flat lesions and lesions with an uninterrupted fifth later, whereas the endoscopic R0 resection rate was low for non-flat lesions. Although the number of each lesion type was not large, we considered that the numbers were acceptable to suggest an association between these findings and resectability. However, these results should be confirmed in a larger study. Moreover, salvage treatment following endoscopic R1 resection should be considered. Although esophagectomy is a highly curative treatment, it has high morbidity and mortality rates. The efficacy of other treatment options such as PDT should be evaluated along with no additional treatment.

Our study had several limitations. First, this was a single-center, retrospective study. Second, EUS was not performed before ER for all non-flat lesions. Third, lesions treated by PDT or esophagectomy were excluded. However, there are ethical concerns for performing ER when the lesion is difficult to completely resect by ESD or EMR or when patients refuse to undergo ER. Fourth, lymph node or organ metastases after ER and long-term outcomes were not evaluated in this study. However, the main aim of this study was to identify endoscopic and EUS findings associated with the complete endoscopic removal of local residual/recurrent cancer.

In conclusion, regarding the treatment for local residual/recurrent cancer after CRT/RT, flat lesions on conventional endoscopy and lesions with an uninterrupted fifth layer on EUS are good candidates for ER.

## Data Availability

The dataset analyzed during this study are not publicly available because of individual privacy. They are available from the corresponding author on reasonable request.

## References

[1] Bray F (2018). Global cancer statistics 2018: GLOBOCAN estimates of incidence and mortality worldwide for 36 cancers in 185 countries. CA Cancer J. Clin..

[2] Rustgi AK, El-Serag HB (2014). Esophageal carcinoma. N. Engl. J. Med..

[3] Kato H (2009). A phase II trial of chemoradiotherapy for stage I esophageal squamous cell carcinoma: Japan Clinical Oncology Group Study (JCOG9708). Jpn. J. Clin. Oncol..

[4] Taniyama Y (2018). Different strategy of salvage esophagectomy between residual and recurrent esophageal cancer after definitive chemoradiotherapy. J. Thorac. Dis..

[5] Sugawara K (2019). Association of preoperative inflammation-based prognostic score with survival in patients undergoing salvage esophagectomy. Dis. Esophagus.

[6] Hatogai K (2016). Local efficacy and survival outcome of salvage endoscopic therapy for local recurrent lesions after definitive chemoradiotherapy for esophageal cancer. Radiat. Oncol..

[7] Ego M (2021). Long-term outcomes of patients with recurrent squamous cell carcinoma of the esophagus undergoing salvage endoscopic resection after definitive chemoradiotherapy. Surg. Endosc..

[8] Kondo S (2016). Prognostic factors for salvage endoscopic resection for esophageal squamous cell carcinoma after chemoradiotherapy or radiotherapy alone. Endosc. Int. Open.

[9] Ishihara R (2008). Local recurrence of large squamous-cell carcinoma of the esophagus after endoscopic resection. Gastrointest. Endosc..

[10] Esaki M (2006). Probe EUS for the diagnosis of invasion depth in superficial esophageal cancer: A comparison between a jelly-filled method and a water-filled balloon method. Gastrointest. Endosc..

[11] Inoue H (1993). Endoscopic mucosal resection with a cap-fitted panendoscope for esophagus, stomach, and colon mucosal lesions. Gastrointest. Endosc..

[12] Oyama T (2012). Counter traction makes endoscopic submucosal dissection easier. Clin. Endosc..

[13] Japan Esophageal S (2017). Japanese classification of esophageal cancer, 11th edition: Part I. Esophagus.

[14] Kanda Y (2013). Investigation of the freely available easy-to-use software 'EZR' for medical statistics. Bone Marrow Transplant.

[15] He LJ (2014). Endoscopic ultrasonography for staging of T1a and T1b esophageal squamous cell carcinoma. World J. Gastroenterol..

[16] Kitagawa Y (2019). Esophageal cancer practice guidelines 2017 edited by the Japan Esophageal Society: Part 1. Esophagus.

[17] Park CH (2020). Clinical practice guideline for endoscopic resection of early gastrointestinal cancer. Clin. Endosc..

[18] Ishihara R (2021). Assessment of the diagnostic performance of endoscopic ultrasonography after conventional endoscopy for the evaluation of esophageal squamous cell carcinoma invasion depth. JAMA Netw. Open.

[19] Hombu T (2018). Salvage endoscopic resection (ER) after chemoradiotherapy for esophageal squamous cell carcinoma: What are the risk factors for recurrence after salvage ER?. Dig. Endosc..

[20] Fukuda H (2020). Effect of horizontal margin status and risk of local recurrence after endoscopic submucosal dissection for superficial esophageal cancer. JGH Open.

